# Advances in Rodent Models for Breast Cancer Formation, Progression, and Therapeutic Testing

**DOI:** 10.3389/fonc.2021.593337

**Published:** 2021-03-26

**Authors:** Chong Liu, Pei Wu, Ailin Zhang, Xiaoyun Mao

**Affiliations:** ^1^ Department of Breast Surgery, The First Affiliated Hospital of China Medical University, Shenyang, China; ^2^ Department of Surgical Oncology, The First Affiliated Hospital of China Medical University, Shenyang, China

**Keywords:** mouse model, mouse intraductal model, transgenic mouse model, breast cancer, rodent model

## Abstract

Breast cancer is a highly complicated disease. Advancement in the treatment and prevention of breast cancer lies in elucidation of the mechanism of carcinogenesis and progression. Rodent models of breast cancer have developed into premier tools for investigating the mechanisms and genetic pathways in breast cancer progression and metastasis and for developing and evaluating clinical therapeutics. Every rodent model has advantages and disadvantages, and the selection of appropriate rodent models with which to investigate breast cancer is a key decision in research. Design of a suitable rodent model for a specific research purpose is based on the integration of the advantages and disadvantages of different models. Our purpose in writing this review is to elaborate on various rodent models for breast cancer formation, progression, and therapeutic testing.

## Introduction

Breast cancer is the most commonly diagnosed cancer and one of the most common cause of cancer death in women worldwide. Advancement in treatment and prevention of breast cancer lies in elucidation of the mechanism of carcinogenesis and progression. Rodent models of breast cancer have developed into premier tools for breast cancer research, and they have generated important insights into the mechanisms that underpin development of the disease and interventional therapies. This review summarizes various rodent models for breast cancer formation, progression, and therapeutic testing.

## A Brief History of Rodent Cancer Models

In the past century, rodent models have proved to be powerful tools in improving knowledge of the underlying mechanisms and genetic pathways of breast cancer and have also created approaches for modeling clinical tumor subtypes and developing innovative cancer therapies. Certain mouse lines can naturally develop breast cancer, or they can be transplanted with breast cancer. Tumors can also be induced by manipulating the mouse genome or by delivery of a viral infection or carcinogen. The relatively low cost of mice and their high reproductive cycle of only 9 weeks make them excellent models for cancer research. In 1911, the first transplantable mouse mammary tumor line and the epithelial origin of a spontaneous mammary tumor were described by Haaland (1). Jacksons Laboratories showed that a retrovirus caused a high incidence of mammary tumors in mice in 1936 (2). The first xenograft breast cancer model was reported in 1962 *via* the heterotransplantation of human breast cancer into an immune-deficient mouse (3). The development of genetically engineered animal models offered a great leap in understanding the genetic basis of breast cancer. The first report of a transgenic mouse model of breast cancer, Oncomouse. In 1984, The Philip Leder research group generated transgenic mice using mouse mammary tumor virus (MMTV)/c-myc fusion gene expression. The mice developed mammary adenocarcinomas spontaneously (4), and 3 years later, they produced transgenic mice with coexpression of MMTV/v-Ha-ras and MMTV/c-myc genes, which resulted in a dramatic and synergistic acceleration of tumor formation (5). These milestones established an entirely new research tool with which to explore the genetic processes of breast cancer. The first transgenic mouse model (GEMM) of HER2-positive breast cancer, reported in 1988, represented another milestone in breast cancer research (6). In 1999, Chuxia Deng and colleagues succeeded in developing a mouse model that ablated BRCA1 specifically in mammary epithelial cells, resulting in mammary tumors (7). This mouse model offered a notably large amount of information that greatly facilitated our understanding of the gender- and tissue-specific tumor suppressor functions of BRCA1 and enriched insights into applying these preclinical models of disease to breast cancer research. However, the GEMM requires time-consuming and expensive work, and another main drawback is that it is highly difficult to control the spatial and temporal expression of a gene of interest. Actually, most human breast cancers are not due to hereditary mutations, rather they arise from normal cells that later suffered spontaneous mutations. The technique of virus-mediated gene transfer into selected mammary cells (such as stem cells and specific progenitor cells) at selected times can overcome many of the shortcomings of the existing mouse models and more closely mimics human breast cancer formation in which only one or a few cells mutate to initiate cancer development (8).

## Transplantable Mouse Models for Breast Cancer

Human cancer cells can be grown as transplants in mice. These transplantable models are simple but have been proven to be highly useful for assessment of breast cancer features, biological behaviors, metastatic potential, and response to therapy.

### Cancer Cell Line Transplantation Mouse Model

Cell-derived xenograft (CDX) of human breast cancer is performed from aggressive cancer cell lines. The CDX model from different tumor cell lines has unique characteristics, including relatively homogenous histological features, molecular subtype, and metastatic potential, among other features. The CDX model makes it possible for different mammary cancer cell lines to be transferred to the mouse in a short time, allowing validation of the target genes of interest and the possibility of research on metastasis and therapy response. It represents a relatively homogenous mass but cannot mimic the heterogeneity or the tumor microenvironment of human breast cancer (9). This technique is usually performed in nude mice (deficient in T-cell function) or other immunocompromised mice but cannot mimic the immune system reaction. If the cancer cells are derived from mouse, they can also be grafted into mice with an intact immune system (10, 11). And the long-term growth *in vitro* can alter some features from its origin cell. Triple-negative breast cancer cell lines such as MDA-MB-231, MDA-MB-435, and SUM1315 can be used to generate stable orthotopic or spontaneous metastasis models of breast cancer *via* orthotopic injection in the mammary fat pad (12). The metastatic MDA-MB-231 and SUM149 CDX models can also be generated by injection into the mouse tail vein (13). Not all breast cancer cell lines from human can be used to successfully establish a CDX mouse model (14). ER-positive luminal A cell lines such as T47D or MCF-7 can only form a tumor mass in immunodeficient mice supplemented with subcutaneous estradiol pellets (15), which produces 18–40 times the physiological levels of estrogen in mice (16, 17). HER2 subtype cell lines such as SKBR3 and MDA-MB-453 cells have poor tumorigenic potential (18).

### Patient-Derived Tumor Xenograft Mouse Model

Due to the limitation of CDX models, the patient-derived xenograft (PDX) is generated by xenografting fresh human tumor biopsies that recapitulate the diversity of breast cancer into host mice. This model reflects the tumor original behavior, histopathology, drug response, and metastatic potential of the original tumor (19). In brief, human tumor fragments or tumor cell suspensions are implanted into the immunocompromised mice and subsequently passaged through several generations in mice. The more heavily immunocompromised mice are usually used to generate PDX, such as NOD-SCID mice (deficient in T-cell and B-cell functions) and NSG mice (deficient in T-cells, B-cells and NK cells).

The PDX modes are relatively stable after the third passage in mice (20) and have relatively stable biological behaviors, such as gene expression profiles, intrinsic phenotypes, and genomic alteration, that are similar to the source of human breast cancer (21–24). PDX also has selected structural variation or mutation differences with the original primary tumor, perhaps due to the adaption to transplantation into the new microenvironment (25). PDX models appear to retain the heterogeneity of the parental tumor of origin and experience a “bottlenecking” clonal selection during transplantation and serial passaging (26). Ding et al. reported comprehensive genomic sequence analysis of DNA samples from an African-American patient with basal-like breast cancer for peripheral blood, the primary tumor, a brain metastasis, and a xenograft derived from the primary tumor (25). That group found that the PDX derived from the primary tumor contained all of the primary tumor mutations and displayed a mutation enrichment pattern that resembled the metastasis. The metastatic subclone was present within the primary tumor, the aggressive subclone with clonal selection in PDX. The PDX drug screening test can mimic and predict drug efficacy, especially in triple negative breast cancer (27), and is a pivotal preclinical tool for evaluating drug response and study of the clonal evolution of tumors and new biomarkers (15). TNBC tumors and to a lesser degree the HER2+ tumors, readily adapt to growth in mice, whereas only 2.5% of ER+ tumors successfully formed stable patient-derived breast cancer xenografts (28). PDX models can’t mimic the immune system and tumor-host interaction because it must also be grown in immunocompromised mice.

### Mouse Intraductal Model (MIND) for Studying Cancer Progression and Immunotherapy

The breast ductal system is a complex series of branching tubules extending from intralobular ductules to the major lactiferous ducts that terminate in the nipple. The mouse intraductal model is based on the special structure of the mammary mouse gland. Human cancer cells can be introduced directly into the mouse mammary ducts in immunodeficient mice to mimic the natural progression of cancer cells in the mammary microenvironment. Behbod et al. established the ductal carcinoma *in situ* (DCIS) model by injecting the human DCIS cell lines (MCF-10 and SUM-255) into mouse mammary ducts *via* up-the-teat injection (29). This approach mimicked breast tumor carcinogenesis and progression from *in situ* to invasive disease and spontaneous metastasis to the relevant sites. In contrast to fat-pad-grafted ER+ tumor cell lines that require estrogen supplement, the MIND of MCF-7 achieved a high engraftment rate without hormone supplements and recapitulated the histopathology and kinetics of human ER-positive tumors (16, 17). These MIND models also often developed bone, lung, and brain metastases, whereas fat pad injection xenografts developed few brain and no bone metastases. The Ki67 indices of MIND MCF-7 tumors were 23%, highly similar to MCF-7 cell lines, and the gene expression signatures are highly similar to the luminal B subtype of clinical samples, as shown by Affymetric microarray and PAM50 (30). For the triple-negative breast cancer mouse model, a fully immunocompetent 4T1-based intraductal model can mimic breast cancer advancement and metastasis to the lungs *via* lymphatic or hematogenous dissemination within 4 weeks (31–33), and it can also disseminate to the liver, brain and kidney (34). 4T1 is a mouse breast cancer line derived from a spontaneously arising mammary tumor in BALB/cfC3H mice (35). The 4T1 MIND models overcome immunosuppression and allow effective immunotherapy research for TNBC (33, 36). This model is predictive, retransplantable, and genomically stable and is an economical and practical mouse model for translational research and study of physiologically relevant hormone action in breast carcinogenesis.

## Carcinogen-Induced Rodent Models

Chemical compounds can induce breast cancer. For example, the carcinogen 7,12-dimethylbenzathracene (DMBA), delivered intragastrically by gavage, can induce mammary adenocarcinomas with several morphological types in mice (37). The induced tumors were luminal-like and mostly ER/PR+ (38, 39). Previous research indicated that estrogen exposure was closely related to elevated breast cancer risk in women (40, 41). The 17β-estradiol-induced mammary cancers highly express ER, PR, and GATA binding protein 3 (42–45), and others such as N-nitroso-N-methylurea (NMU) can induce mouse breast cancer similar to that of low-grade estrogen-receptor positive human breast cancer (46–48). Spontaneous chemically induced mouse models are helpful for investigation of the pathogenesis and therapeutics of breast cancer (49).

## Genetically Engineered Mouse Models of Breast Cancer

Genetically engineered mouse models (GEMM) of breast cancer have supplied a wealth of knowledge for both basic cancer research and translational oncology by introducing DNA into the mouse genome. GEMMs reflect some of the diversity of genetic lesions in human breast cancer. These models include three broad groups: transgenic mouse model, knockout mouse model, and genetic models of breast cancer based on intraductal injection of virus to modify the genes of the mammary cells.

### Transgenic Mouse Model

Transgenic mouse models refer to those which have exogenous DNA integrated in their germline as a consequence of experimental DNA transfer application. The integrated DNA may or may not be derived from the same species as the host genome, it may or may not be targeted to an intended site of incorporation in the genome, and it may or may not encode for a functional gene.

The MMTV-PyMT transgenic mouse is a model that carries the polyoma virus middle T-antigen under the control of the mouse mammary tumor virus (MMTV) promoter. The PyMT is involved in multiple oncogenic pathways that lead to an aggressive tumor phenotype such as Src, Ras, and PI3K (50–54). MMTV-PyMT females develop multifocal, poorly differentiated, highly invasive ductal carcinoma as early as 4 weeks of age, reaching the maximum tumor burden at 12 weeks of age, and they also exhibit lung metastasis near 10 weeks of age (55–59). This model is used in breast cancer research to analyze the mechanism of carcinogenesis and alter the tumor microenvironment. Maglione also reported that atypical lesions had levels of detectable ER expression, and the mammary intraepithelial neoplasia and tumor cells had variable sporadic ER-positive nuclei staining (58). Previous research indicated that the PyVT mammary tumors were shown to be ER+, PR+, P53+, and HER-2+ *via* immunohistochemistry at the early stage of tumor formation, progressing to the triple-negative subtype (57, 58). This model has drug resistance to cisplatin and paclitaxel, but tamoxifen is effective in the prestage and early stage of tumor formation (57).

The Wnt-1 (int-1) proto-oncogene was originally cloned following activation by MMTV insertion in mouse mammary tumors (60, 61). The MMTV-wnt-1 mouse model was established with the MMTV-LTR upstream of wnt1 insertion in the opposite transcription orientation (62, 63). This model is characterized by grossly ductal hyperplasia with extensive fibrosis, and these mice can develop breast cancer at an onset of 24 weeks (64). Females cannot deliver milk to their young because of extensive ductal hyperplasia (64). The tumors in MMTV-wnt-1 transgenic mice are composed of myoepithelial (basal-like) and luminal epithelial cells. β-catenin is an integral player in the Wnt signal transduction pathway, and β-catenin transgenic (MMTV‐βcatΔN) mice exhibit mammary gland hyperplasia and mammary adenocarcinoma, which are highly similar to the corresponding lesions in MMTV-wnt-1 mice (65). Wnt10b is a ligand that activates the canonical Wnt/β-catenin pathway, and MMTV-Wnt-10b transgenic mice showed hyperplastic mammary development involving highly branched mammary ducts and gynecomastia (66). LRP6 is a Wnt signaling coreceptor, and MMTV‐LRP6 mice exhibit significant hyperplasia with upregulated expression of Axin2, Cyclin D1, and c-Myc (67). MMTV-c-Myc and MMTV-int2 mice also develop pronounced mammary hyperplasia and adenocarcinoma in proportion (65, 68). Indeed, the wnt-associated mouse model has made a great contribution to elaboration of the wnt pathway in breast carcinogenesis.

The first MMTV-ErbB2 transgenic mouse model expressed an activated Erbb2 under promoter of MMTV-LTR, and these mice are viable and fertile (6). There is no phenotypic effect in males. This transgene is expressed at low levels in the normal mammary epithelium, salivary gland, and lung (69, 70), and higher expression is detected in tumor tissue. This model produces multifocal and stochastic mammary tumor formation near 15 weeks of age (69, 71) and lung metastasis with long latency (approximately 32 weeks or longer) (72) and had positive cyclin D1 and CDK4 expression and a high Ki-67 proliferative index. In contrast to the MMTV-ErbB2 mouse line, Muller et al. later established transgenic mice carrying unactivated neu under the MMTV promoter (73). The mice began to develop focal mammary adenocarcinoma surrounded with hyperplastic mammary epithelium at 16 weeks of age, with decreased neu intrinsic tyrosine kinase activity. Many of these tumor-bearing transgenic mice with unactivated neu also developed metastatic tumors in the lung (73). Li et al. found that 37% of tumors in the MMTV-ErbB2 mouse had mis-sense mutations in p53 (74), and thus, they established bitransgenic mice carrying MMTV-neu and a 172Arg-to-His p53 mutant (p53-172H). The bitransgenic mice developed anaplastic and aneuploidy tumors that exhibited increased apoptosis, distinct from the tumors with diminished apoptosis arising in p53-null mice (74).

The C3(1)/SV 40/t-antigen (C3(1)/Tag) mouse model contained a recombinant gene expressing the simian virus 40 early-region transforming sequence under rat prostatic steroid binding protein [C3(1)]. Female hemizygous mice generally developed mammary hyperplasia at 9 weeks of age and mammary intraepithelial neoplasia with similarities to DCIS at 12 weeks, with subsequent development of mammary adenocarcinoma at an onset of 24 weeks in 100% of the animals and 15% incidence of lung metastasis (75–81). This model develops invasive carcinoma independently of hormones or pregnancy (72). All mammary adenocarcinomas were diffuse immunopositive for CK14, CK18, and p53 and negative for αSMA, ERα, PR, and C-erbB-2 (81). Previous study indicated that human basal-like breast cancer exhibits a high frequency of p53 mutation of deletion. It is a suitable mouse model for research on basal-like breast cancer because of the gene expression and DNA somatic alteration levels.

Cyclin D1 is essential in breast carcinogenesis induced by c-neu and v-Ha-ras and not induced by c-myc or Wnt-1 (82). The MMTV-cyclin D1 mouse can develop mammary adenocarcinomas quite late stochastically (83, 84). Cyclin E is a cancer marker that is the limiting factor for the transition from G1 to the S-phase of the cell cycle, which determines ignition of DNA duplication. Previous research indicated that the 27% low-molecular-weight isoform of cyclin E transgenic mice can induce metastatic mammary carcinoma (85).

### Knockout Mouse Models of Breast Cancer

Knockout animals are mice with targeted disruption of selected endogenous gene sequences. These models are used to reveal valuable clues related to the biological and molecular function of a gene in the setting of a developing or developed tumor. The constitutive knockout model refers to the whole-body knockout model, i.e., the target gene is knocked out in all tissues at all times. Many tumor suppressors often result in lethality during embryonic development or at developmental stages prior to tumor formation. This obstacle has been effectively overcome by applying the conditional knockout model (86) in which the gene knockout can be spatially and even temporally regulated. With a conditional KO, gene inactivation can occur in a certain tissue type, made possible by Cre-LoxP and Flp-Frt recombinase system. Today, the development of the clustered regularly interspaced short palindromic repeats (CRISPR)/Cas9 technique has made conditional knockouts even more popular and widely used. This new technology is more efficient and easier than the Cre-LoxP or Flp-Frt recombinase technology. Therefore, we summarize the tumor phenotype of the popular conditional knockout strains reported in the literature.

BRCA1 inherited mutations predispose carriers to female breast and ovarian cancers. Constitutive knockout of mouse BRCA1 causes recessive mouse embryonic lethality (87), and therefore, the BRCA1 conditional mutant mouse model was used to overcome this obstacle (88). Exon 11 is a large central exon of 3426 bp that represents 60% of the coding sequence in BRCA1 (89). In 1999, Xu established a BRCA1flox11 mutant mouse, which was achieved by deleting only exon 11 of the full-length BRCA1 gene and leaving expression of the short BRCA1 transcript with loxP sites (BRCA1flox11) (7). The 25% BRCA1flox11 mutant mouse develops mammary tumors after a long latency (7). The 94% BRCA1flox11 mouse develops mammary tumors with a long latency (T50 = 17 months), and the tumors exhibit an atypical medullary phenotype strongly reminiscent of basal-like breast tumors (90). Xu et al. found that the BRCA1flox11 mutation mouse often had had spontaneous p53 mutation, and thus they introduced heterozygous deletion of p53 in the BRCA1flox11 mouse, which accelerated tumor formation (91). Weaver et al. also revealed that certain of the tumors had structural abnormalities on the map location of c-myc gene, Rb1, and p53, similar to BRCA1-associated breast cancer in patients (92).

Other conditional BRCA1 alleles are reported to cause functionally null BRCA1 alleles by flanking exon 2 (BRCA^f2^) (90), exons 5–6 (BRCA1^f5–6^) (93), exons 5–13 (BRCA1^f5–13^) (94), or exons 22–24 (BRCA1^f22–24^) (95). The BRCA1^f5–13^ mouse had intermediate to high grade tumors with high mitotic count, expansive growth, moderate to high nuclear grade also displayed ER-negative immunohistochemistry staining with pushing borders, and increased expression of basal epithelial markers, similar to human basal-like breast cancer (94). The 64% mouse with BRCA1^f22–24^ mutation combined with heterozygosity for a p53 mutation developed tumors with basal-like markers in all cases before 22 months of age. This model had high histological grade, central necrotic areas, and presence of homologous metaplastic elements and is a suitable model for metaplastic basal-like breast cancers (95).

Germline mutations of BRCA2 are associated with one-third of hereditary breast cancer. Jonker et al. generated a mouse model with conditional mutants of BRCA2^f11^ (flanking exon 11 of the gene with loxP sites) and found that no BRCA2^f11^ mice developed tumors. The mammary glands and skin frequently developed tumors in females carrying conditional BRCA2^f11^ and p53 knockout alleles (96). The vast majority of the mammary tumors were carcinomas with myoepithelial or basal cell types. Most tumors arising in the conditional BRCA2^f11^ and p53 knockout mice had high-grade invasive ductal carcinoma, with a solid growth pattern, a large CK8-positive and ER-negative cell type with high mitotic count, high-grade nuclei and with pushing borders (96). The tumors often harbor the undifferentiated basal cell type. Based on the results from cross-species comparison by unsupervised clustering, these tumors are closely similar to human BRCA1-mutated breast cancers with basal-like phenotypes. Ludwig generated mice with BRCA2^f3–4^ (flanking exons 3 and 4 of the gene with loxP sites) mutation, which had a high incidence (77%) of breast tumors that developed in one or more glands after a long latency (time for median tumor-free survival of approximately 1.4 years; total of 40 tumors in 20 animals) (97). In addition, Cheung generated a mouse model with BRCA2^f9–11^ (flanking exon 9–10 of the gene with loxP sites), which had mammary adenocarcinomas after a long latency (average, 1.6 years). A subset of these tumors also showed downregulated p53 expression (98).

As mentioned previously, the p53 mutation is linked tightly with breast cancer. The conditional knockout p53^Δ2–10^ (deletion of exon 2–10 of the gene with Cre recombinase) model generated by Jonker et al. develops lymphomas and sarcoma rather than epithelial tumors (96), and therefore, those researchers crossed p53^Δ2–10^ mice with K14-cre transgenic mice (Cre recombinase expression is restricted to skin and mammary gland epithelium and other epithelial tissues). The resulting K14-cre p53^Δ2–10^ mice developed mammary tumors with a median latency (T50) of 288 days. Interestingly, 38% of the mammary tumors were pure epithelial tumors (intermediate to high-grade), 48% were poorly differentiated biphasic carcinoma, and 14% were well differentiated biphasic carcinoma. The molecular signatures of these tumors showed a significant association with human sporadic ER-negative tumors (94). These tumors closely mimic human sporadic basal-like breast cancer. Lin et al. generated a mouse breast cancer model with inactivated p53 (deletion of exon 2–10 of the gene with Cre/loxp) in mammary epithelial cells (99). The tumors are heterogeneous, including adenocarcinoma, myoepithelial adenocarcinoma, spindle cell tumor, and adenosquamous carcinoma, and most were poorly differentiated invasive adenocarcinomas, which share the most histopathological similarity with human breast cancer. A total of 35% had c-myc amplification, and 66% had erbB2 overexpression. The tumors were initially ERα-positive but progressed to ERα-positive and -negative tumors (99), similar to human breast cancer. Multistep histopathological changes and alterations in the ERα expression pattern are observed during progression of mammary carcinogenesis in these models.

PTEN is a tumor suppressor that is frequently mutated in breast cancers. Germline PTEN mutations cause inherited syndromes that lead to an increased risk of breast cancer. Wu Hong and colleague generated PTEN^Δ5^ allele (flanking exon 5 of PTEN with loxP sites) and established mammary-specific PTEN deletion mice (100, 101). PTEN null mammary epithelial cells were hyperproliferative and showed decreased apoptosis. Mutant females developed mammary tumors with upregulated populations of cells expressing cytokeratin 5 and 6 within 400 days (101). When a PTEN conditional allele was mated with MMTV-NIC mice, which coupled expression of Cre and activated ErbB2 from the bicistronic transgenic transcript, all female mice developed multifocal mammary tumors and high lung metastases, which displayed histopathological and molecular characteristics of the luminal subtype of primary human breast cancer (102).

### Genetic Models of Breast Cancer Based on Intraductal Injection of Virus for Delivery of Oncogenic Mutations to Mimic Human Cancer Formation

**Figure 1:**
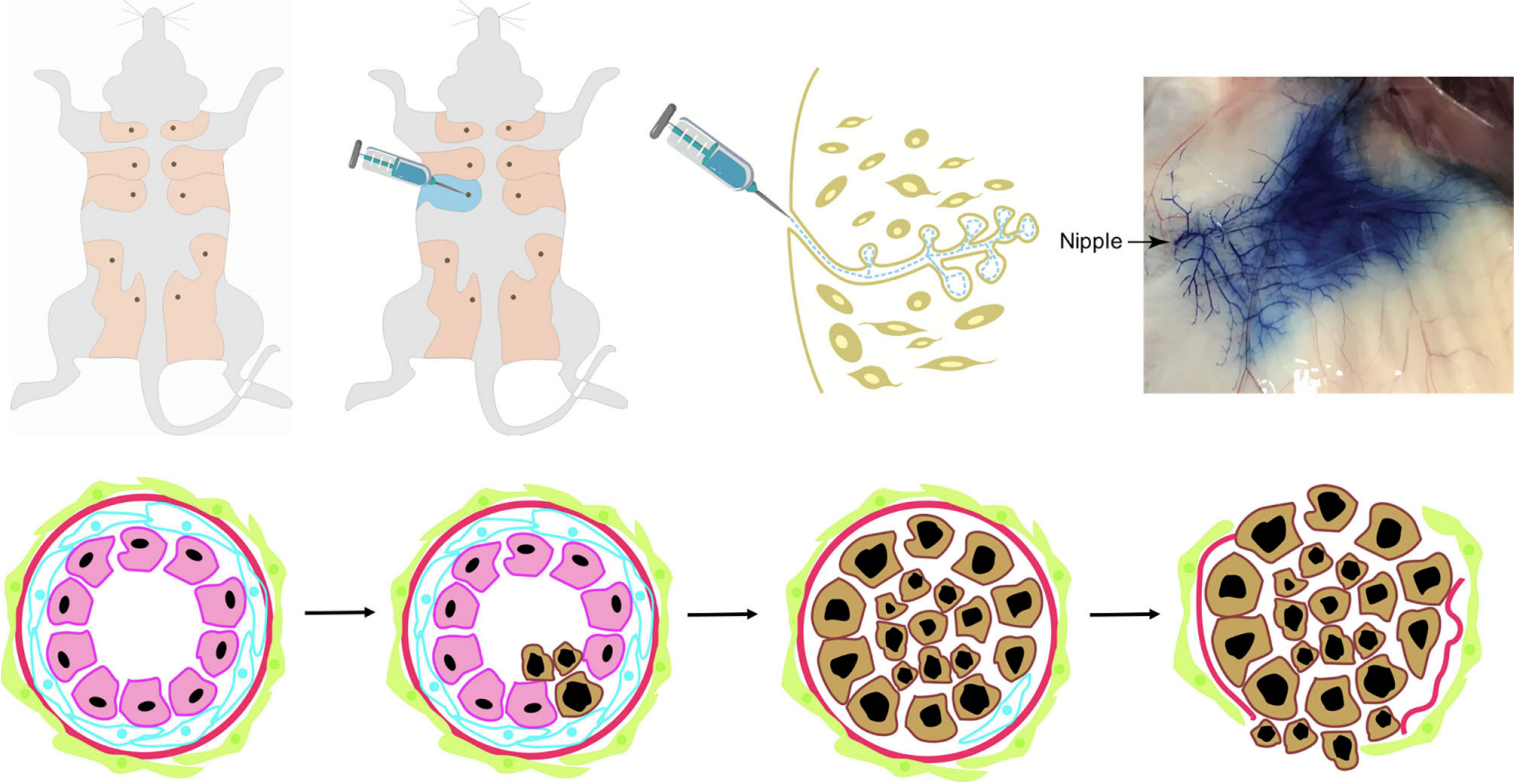
The intraductal injection of retrovirus into mammary glands *in vivo* with virus vector initiates and promotes carcinogenesis in mouse models. It is a novel mouse mammary gland cancer model which mimics human breast cancer non-invasive-to-invasive progression with virus vector. Most human breast cancers are not due to hereditary mutations, rather they arise from normal cells that later suffered spontaneous mutations. This mouse model was established by intraductal injection of retrovirus carrying the oncogenes with blue dye into one of the fifth mouse mammary glands. The technique of virus-mediated gene transfers into selected mammary cells (such as stem cells and specific progenitor cells) at selected times can overcome many of the shortcomings of the existing mouse models and more closely mimics human breast cancer formation in which only one or a few cells mutate to initiate cancer development. It allows temporal analysis of many processes involved in early breast cancer invasive progression including intraductal cancer cell growth, the cell interactions with the surrounding normal epithelial and myoepithelial cells, and their escape into the surrounding stroma. Photo of nipple injection -- courtesy of Wen Bu (Baylor College of Medicine).

Based on molecular biology, breast cancer is highly complicated. Most human cancers are not due to hereditary mutations, and instead, they arise from normal cells that later suffer spontaneous mutations. It is notably difficult to manipulate the spatial and temporal expression of genes in mouse. Genetic models of breast cancer based on intraductal injection of a virus can circumvent selected disadvantages of the typical transgenic or knockout mouse models. Currently, in clinical and basic research, compound techniques of mouse models have more practical applications. The avian leukosis-sarcoma virus (ALSV) and its specific receptor tumor virus A (TVA) play a vital role in this model. Mammalian cells lack the TVA gene sequence, and the transfer of the TVA gene to specific cells in mouse renders them uniquely susceptible to infection by ALSV-based RCAS virus (103). RCAS viruses can be delivered into mice by injection of virus-producing cells or by injection of concentrated virus (103, 104). Harold Varmus and colleagues constructed the RCAS-TVA avian retroviral system, which can carry oncogenes (e.g., K-ras, c-myc), marker genes (e.g., green fluorescent protein, alkaline phosphatase), dominant negative tumor suppressors (e.g., mutant p53), or recombinases (e.g., Cre) (105). This method offers a precise way to manipulate the temporal and spatial expression of genes in the mammary epithelium. A single TVA mouse stain can be used to evaluate the effects of multiple genes, individually or in combination, instead of generating a mouse line for each gene of interest. Yi Li modeled breast cancer in a mouse with the RCAS-TVA system by mammary gland intraductal injection (106) (**Figure 1**). Precancerous lesions can be detected by 7 days following RCAS-PyMT injection (107). The PyMT oncogene delivered by RCAS-TVA caused multifocal mammary tumors after a notably short median latency of only 12.5 days. The tumors were composed of myoepithelial (basal-like) and luminal epithelial cells and were relatively well differentiated, consisting of many acini and heterogeneous cell types with ER positive expression (8, 108). In mice injected with RCAS-erbB2, precancerous lesions can be detected 14 days after injection (109). The mice developed high grade, poorly differentiated, stroma-rich, and ER-negative mammary tumors (109–111).

## Challenges in Modeling ER+ Breast Cancer

Majority GEMMs are ER negative and most xenograft mouse models are based on few ER+ cancer cell lines (112). And there are no reliable mouse models of ER+ breast cancer that are also estrogen-dependent (113, 114). For example, STAT1−/− mice express abundant amounts of ER and PR (115), but tumor development is not hormone-dependent (116). A K-Ras mutant has been reported to induce ER+ tumors in mice (114), but the resulting tumors have not been thoroughly tested for estrogen dependency. As we previous indicated that the RACS-TVA approach can especially introduce genetic alterations into only a small number of the mammary cells (103). Lentiviral PyMT produces both luminal and basal-like tumors (55). TVA-PyMT mice and TVA-erbB2 mice had ER expression in greater than 10% of mammary tumor cells (117). And PyMT-induced tumors exhibited a two-fold increased ER-positivity *versus* erbB2-induced tumors. Compare with mice mammary glands, rats are more similar to the human breast, rats mammary glands had a ductal tree terminates in TDLUs with connective tissues and organized fibroblasts as sheath around and shows extensive alveolar development (118). Oral DMBA or intravenous or subcutaneous of NMU induced ER+ and PR+ tumors in rats (119), and many of these tumors harbor Ras mutations (49, 120). Ras signaling is frequently activated in human breast cancer, usually not by mutations in a Ras gene per se, but by mutations and overexpression of upstream signaling components such as receptor tyrosine kinases and NF1 mutations (121). Wang et al. found that intraductal injection of retrovirus expressing activated versions of Ras or erbB2 into Sprague/Dawley rats led to ER+ tumors (122). This intraductal model has a defined genetic mutation and is more relevant to human breast cancer etiology than DMBA models. NF1 mutations are enriched in ER+ breast cancers of patients. Crispr-mediated germline knockout of NF1 has been reported to induce ER+ tumors that are estrogen dependent (123). The CRISPRs technology is already widely used to edit somatic cells, and CAS9 rats are already commercially available. Therefore, intraductal injection of a virus carrying gRNA could be used to mutate NF1 and other genes associated with human ER+ cancer to generate somatic models of ER+ cancer in rats. Wen and Yi also described the intraductal injection of lentiviral vector FUCGW carrying the mutated oncogene HrasQ61L to Sprague/Dawley rats led to mammary tumors with high positive expression of both ER and PR (124). This technique is an efficient tool for modeling formation, prevention, and treatment of human breast cancer, especially ER+ breast cancer.

## Translational Application of Rodent Models for Breast Cancer Treatment

Actually, to mimic human breast cancer accurately is very difficult, especially in breast cancer therapy. CDX or PDX models are widely used because of its easy application, large and rapid tumor cohort generation, and simple preclinical data assessment. They can’t recapitulate tumorigenicity and treatment response in immunocompromized or immune-competent host system. In clinic, cyclin dependent kinase 4/6 (CDK4/6) inhibitors PD0332991 (palbociclib) has shown great efficacy in the treatment of hormone receptor-positive breast cancer, has received conditional approval from the FDA for metastatic breast cancer. Roberts et al. indicated that palbociclib is effective in a HER2-positive mouse model of breast cancer (MMTV-c-neu) but had no effect in the basal-like breast model C3-TAg (125, 126). The combination of carboplatin plus PD0332991 decreased antitumor activity compared with carboplatin alone in MMTV-c-neu with hematopoietic stem cell dormancy (125). It mimicked the therapy response of palbociclib in different subtype breast cancer. Usary et al. examined a range of therapeutics focused on MEK, mTOR, and PIK3CA/mTOR inhibitors in basal-like (C3-TAg), luminal B (MMTV-c-neu), and claudin-low (T11/TP53*^−/−^* OST) GEMM (127). They found variable responses in different GEMM. The MMTV-c-neu and basal-like breast model C3-Tag was the most responsive in general and claudin-low T11/TP53−/− model was the most resistant with only small responses. GEMMs recapitulated characteristics of human breast cancer have become a promising tool in cancer research to predict clinical outcome. A successful GEMM is very slow and laborious so that it has not been widely used. And there are still a lot of deficiencies with GEMM in preclinical research. The “co-clinical” trials which are validated *in vivo* models to pursue high-throughput drug screening and rapid translation of effective anticancer drugs into the clinical setting (128, 129). The co-clinical trials are underway in breast cancer, and we are looking forward to better rodent models for therapeutic testing of breast cancer.

## Summary

The selection of appropriate rodent models for investigation of breast cancer is an important experimental decision. Every mouse model has advantages and disadvantages, and thus it is highly important to design a suitable mouse model for each research purpose based on integration of the advantages and disadvantages of different models, and compound techniques of mouse models have more practical application. The rodent models may help to improve the knowledge of breast carcinogenesis mechanism and genetic pathways, as well as creating therapy for modeling clinical breast cancer subtypes and develop innovative cancer therapy.

## Author Contributions

All authors made substantial contributions to articles reviewed in this manuscript, were involved in the drafting and revision, and approved the final version of this manuscript. All authors contributed to the article and approved the submitted version.

## Funding

This work was supported by the National Natural Science Foundation of China (No. 81972791). The funders had no role in study design, data collection and analysis, decision to publish, or preparation of the manuscript.

## Conflict of Interest

The authors declare that the research was conducted in the absence of any commercial or financial relationships that could be construed as a potential conflict of interest.
